# Chronic Obstructive Pulmonary Disease: The Present and Future

**DOI:** 10.3390/biomedicines10020499

**Published:** 2022-02-20

**Authors:** Aditya Krishnan, Alice M. Turner

**Affiliations:** Department of Respiratory Medicine, University Hospitals Birmingham NHS Foundation Trust, Birmingham B9 5SS, UK; a.m.turner@bham.ac.uk

## 1. The Present of COPD

Chronic obstructive pulmonary disease (COPD) is a highly prevalent condition associated with smoking and is predicted to become a leading cause of death in the current decade [1]. The condition is characterised by small airway inflammation and progressive parenchymal destruction, resulting in loss of lung tissue and obstructive pulmonary dysfunction due to gas trapping, poor expiratory flow and impaired gas exchange [2].

**Figure 1:**
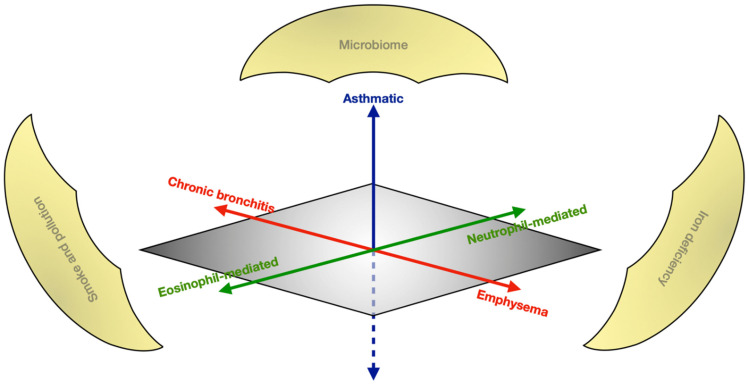
The heterogeneous spectrum of COPD. The x-axis (red) demonstrates the radiological dichotomy of characterising syndromes in COPD; the y-axis (blue) represents the nosological complexity of COPD’s physiologic overlap with asthma; and the z-axis (green) shows the cellular mediation driving the disease. Overarching environmental factors (gold) perpetuate the disease.

COPD is a nosologically complex disease known to exhibit widely heterogeneous phenotypes requiring personalised approaches to management. Figure 1 describes the varied radiological, physiological and cellular characteristics defining COPD, and highlights their interactions with environmental factors. Whilst smoking and ageing are established as principal risk factors, COPD is multifactorial with genetic predisposition and interacting environmental triggers, the latter of which unlock epigenetic predispositions. There are poorly understood epigenetic mechanisms involving histone modifications caused by microRNA inducing inflammation, causing airway destruction and predisposing to malignant transformation [3]. The goals of treatment in stable-state COPD aim to improve quality of life (QoL), exercise capacity, reduce exacerbations and prevent premature mortality. The management of COPD includes conservative measures such as smoking cessation and exercise, in addition to a number of established pharmacological therapies [4]. COPD is also a significant risk factor for the development of morbidity and mortality, such as through the increased malignancy risk. There is an increasing emphasis on endotype-directed treatment based on the phenotype at presentation [5].

This Special Issue includes various study designs presenting endotype-directed therapies to target interventions towards the right patients; observational studies exploring the complex interactions between comorbidities, COPD, and the therapies used therein; and authors’ calls for novel methods for performing and presenting research to allow us to stratify COPD care in the future.

## 2. Phenotype-Guided Therapies

The basis of precision medicine is that by distinguishing endotypes, we can target therapies towards those who are most likely to benefit, and potentially avoid iatrogenic consequences from unnecessary treatment [6]. There is a paradigm shift in medicine towards using bioinformatics to identify patients with genetic predispositions or biomarkers.

### 2.1. Genetic and Epigenetic Mechanisms

The relationship between COPD and lung cancer is well established. Despite adjusting for smoking history, lung cancer is more prevalent amongst COPD patients than non-COPD smokers [7]. Endotype-specific pathophysiology has been identified in murine models, which have demonstrated the development of emphysema following deliberate endothelial apoptosis. Additionally, the proportion of emphysematous lung is correlated with endothelial dysfunction in COPD patients. Emphysema is independently associated with a five-fold-increased relative risk of developing lung cancer.

An included study by Green et al. compares microRNA targets between tumour and non-tumour lung tissues from a mixture of COPD and non-COPD patients [8]. Green et al. identified that several microRNA targets were upregulated in tumours, including targets previously associated with non-pulmonary smoking-related cancers. The results suggest a mechanistic link of microRNA inducing endothelial apoptosis in COPD, as seen in emphysema. It has been established that there are shared microRNA signatures between COPD and lung cancer, but this is the first paper to describe cellular microRNA differences in expression between patients with and without COPD.

It will be interesting to consider whether intercellular signals interfere with the phenotypic expression of these signatures, as it may be that the effects of these microRNA are not observed at a tissue level. This paper also validates the function of microRNA changes in vitro—this aspect can be used as a foundation for studying the same functions in whole lungs and correlating them with clinical presentations and outcomes. As this study demonstrates in vitro how microRNA overexpression can result in endothelial apoptosis, future therapies could investigate microRNA correction, as has been demonstrated with retinoic acid in murine models [9].

The authors identify an evidence gap in the expression of microRNA in cancer cells with and without established COPD, which a study by Qin et al. successfully fills [10]. Qin et al. isolated serologic microRNA and oxidative stress markers from patients with and without lung cancer, some of whom had COPD. They identified certain tumour-suppressive microRNA to be underexpressed in COPD patients who developed lung cancer, further supporting it as a surrogate marker for tumorigenesis in COPD patients [11]. Furthermore, certain microRNA levels were significantly associated with an enhanced sensitivity to agents such as cisplatin, suggesting a potential for genotype-targeted therapy and prognostication in the future. As anticipated, plasma antioxidants were upregulated in cancer and COPD cohorts, fitting with the existing theory of COPD-driven systemic oxidative stress, which in itself induces carcinogenesis. A previous meta-analysis identified a pathophysiological relationship between markers of oxidation with tumorigenesis in this cohort [12].

Qin et al. describe a number of genotypic and phenotypic changes observed in patients with lung cancer. Specifically, they identify significant differences between lung cancer patients with concurrent COPD compared to lung cancer patients without COPD. Given the statistically similar distribution of active smokers and number of pack years in each described group, this study sidesteps a major confounding variable otherwise observed in similar research. Nevertheless, there remains a large evidence gap between the COPD-mediated oncogenic mechanisms and our understanding of the clinical pathophysiology behind lung cancer.

Finally, although we are aware of inflammatory and immune-mediated pathways in COPD pathogenesis, we are unclear regarding which factors determine whether patients exposed to tobacco will develop the condition. An included review by Carrasco-Hernández et al. outlines the tobacco-associated functional alteration of the cystic fibrosis transmembrane conductance regulator (CFTR) in COPD, resulting in airway dehydration [13]. They describe fascinating research methods that have been applied to COPD, such as using CFTR-deficient mice to build models of smoke-induced COPD. Authors suggest we look to CFTR modulators used in cystic fibrosis to modify the acquired dysfunction in COPD. Related drugs such as ivacaftor are already under study in COPD.

### 2.2. Lung Microbiome

Up to half of all COPD patients are colonised with potentially pathogenic microbes (PPMs) associated with persistent neutrophil-mediated inflammation and accelerated clinical deterioration. *Haemophilus influenzae* has extensively been described as the most common PPM in stable-state COPD and is particularly associated with the neutrophil-mediated endotype. Three papers in this Special Issue describe unique methods of estimating PPM.

A systematic review by Armitage et al. clarifies the prevalence of colonisation in stable-state COPD and identifies its relationship to the modality of sample collection [14]. The authors systematically identified 36 studies, which included a total of over 3000 patients, to perform a meta-analysis, identifying *H. influenzae* as the most prevalent PPM, colonising 41% of samples regardless of sampling method. This systematic review makes use of various methods such as multiple raters to ensure the validity of the analysis. The paper provides a large-scale overview and identifies a high level of heterogeneity in the results, likely due to the variation in defining and reporting PPMs. The authors raise this issue in reporting that various studies identified or set culture conditions based on likely predefined PPMs, potentially underrepresenting other bacteria and frequently omitting fungi.

Another review by Keir et al. describes next-generation sequencing methods that overcome the limitations of the culture mediums described by Armitage et al. [4]. They describe *Streptococcal* species as more common in eosinophil-mediated COPD, and that loss of microbiome diversity is associated with more frequent exacerbations. They identify the dearth of evidence on the direct effects of corticosteroids on bacteria, as most ongoing research is murine or in vitro.

A study by Beech et al. performed sputum quantitative polymerase chain reactions to quantify bacteria in patients with stable-state COPD [15]. Compared to controls, COPD patients had higher quantities of *H. influenzae* than the upper threshold of normal—no other demographic features distinguished this cohort of *H. influenzae*-positive COPD patients. They confirmed that patients with sputum eosinophilia were rarely colonised with any dominant bacterial species at either visit, and especially demonstrated a lower probability of *H. influenzae* colonisation. This is consistent with previous research which suggests that T2-mediated eosinophil-dominant and bacteria-mediated neutrophil-dominant endotypes are mutually exclusive. Researchers also defined bacterial species-specific thresholds from healthy participants to identify “abnormal” levels in COPD patients; for instance, *Streptococci* were abundant in COPD patients, but no more than in healthy volunteers, and so were not implicated in the pathophysiology of COPD.

As frequently seen in longitudinal research, participant attrition is expected. In this study, attrition reduced the available sample size for the pairing of sputum samples and longitudinal analysis of bacterial colonisation. Some demographic variables, such as age and smoking history, could be better matched. Various epidemiological studies over time have concluded that eosinophil-endotype COPD patients are rarely colonised with *H. influenzae*, but the distinct microbiome seen in these patients is yet to be described.

These findings form the basis of studying the consequences of specific microbial colonisation on exacerbation phenotype, opening another avenue for endotype-targeted therapy. The next steps are to understand the interactions involved in co-colonisation and its effects on clinical stability. Disease progression is known to raise the likelihood of colonisation; does colonisation then further contribute to disease progression?

## 3. Comorbidities

Patients with COPD tend to be multi-morbid due to the condition’s strong association with inherited and lifestyle risk factors. Consequently, conditions such as hypertension, dyslipidaemias and atherosclerosis are prevalent amongst COPD patients [16]. Common confounders exacerbate COPD and these comorbidities; COPD and comorbidities exacerbate each other; and therapies used have adverse effects [5]. Cluster analyses demonstrate that the severity of comorbidities plays a role in the progression of pulmonary disease, as well as all-cause mortality [17].

In addition to various sociocultural confounders such as smoking affecting other organ systems, COPD therapies also worsen comorbidities. This is particularly true for inappropriately prescribed corticosteroids in neutrophil-mediated COPD, despite their significant adverse effect burden [18,19].

### 3.1. Functional State

One such comorbidity, which both worsens COPD and is contributed to by COPD, is frailty. A recent meta-analysis estimated a frailty prevalence of one in five amongst COPD patients [20]. These patients are also associated with poorer clinical outcomes and the development of mood disorders. Extrapulmonary manifestations from chronic inflammation and chronic hypoxia worsen small vessel disease, particularly in the vulnerable neuroplastic hippocampus.

Takahashi et al. present an observational study exploring the relationship between hippocampal volume and clinical outcomes in patients with COPD [21]. Frail stable-state COPD patients and those reporting poor QoL were identified to have smaller estimated hippocampal volume via magnetic resonance imaging. This association does not seem to correlate with respiratory function. Arguably, this area requires further research since the authors were unable to establish significant links between respiratory function and hippocampal imaging, despite the possibility of mechanistic links. The study could benefit from the introduction of non-COPD controls to ensure adequate power and consider the impact of comorbidities on quality of life to illustrate a better picture of disease consequence. Future longitudinal studies may help establish an understanding of sequelae and causality. Is disease-driven frailty causing hippocampal pathology in COPD patients, or is chronic inflammation the common root for poor baseline functioning and cerebral changes?

### 3.2. Iron Balance and Metabolism

An emerging area of interest in COPD is its association with iron, due to its pathophysiological role in redox balance and its clinical role in anaemia. Several implicated pathways result in oxidative damage, protease-mediated damage and inflammation in lung parenchyma and airways. Additionally, inflammatory states exacerbate iron deficiency. The correction of iron deficiency demonstrates clinical benefits in non-anaemic patients with conditions such as heart failure [22].

As in the trial by Pérez-Peiró et al., these parameters can be measured biochemically by monitoring indirect markers of oxidative stress and antioxidants [23]. The authors demonstrated a decline in oxidative stress markers in non-anaemic COPD patients randomised to intravenous iron replacement within a short four-week study. Hepcidin was identified as a potential diagnostic and prognostic measure for iron deficiency in COPD.

This is a well-presented single-blinded randomised controlled trial (RCT), which effectively uses violin plots to express findings concisely. These statistically significant findings could form the basis for larger multi-centre trials, strengthening the impact of this paper if attrition characteristics were specified for intervention and control groups. Future research will determine whether the correction of iron deficiency has a clinical benefit beyond biochemical changes. Determining iron status and managing it as an oxidative stressor may be a direction for personalising COPD care.

In addition to the emerging role of systemic iron deficiency, cigarette smoking and COPD are associated with raised lung macrophage iron. These stressors contribute to the development of reactive oxygen species, which drive airway modelling and chronic inflammation. The study by Baker et al. quantified red blood cells (RBCs) and macrophage iron levels in lung tissue samples, confirming a correlation between spirometry and lung macrophage iron [24]. The novel finding is that RBCs are the likely source of iron loading, resulting in dysmorphic macrophages, and thereby raising the risk of bacterial infection. This in vitro study can form the basis for clinically correlated research, investigating other consequences of pulmonary vascular leakage, including chronic inflammation, endothelial damage and venous stasis.

## 4. Epidemiological Findings

As demonstrated, research in COPD is thriving. However, the nature of this disease makes comparisons between trials difficult due to heterogeneous patient groups. As a result, the European Respiratory Society (ERS) publishes consensus statements on core outcome sets that prioritise the most critical outcomes in COPD [25].

### 4.1. Reporting COPD Outcomes

This Special Issue includes a systematic review investigating the instruments used in quantifying COPD exacerbations [26]. They identified almost 200 intervention-based RCTs and reported that studies were using invalidated composite instruments with varied follow-up periods. Studies using prolonged follow-up durations and stricter instruments were more likely to report treatment failure. Future trials may benefit from studying ERS-defined outcomes rather than producing composite scores that overestimate treatment effects. Equally, the work presented on expected treatment failure at various follow-up time periods in this review could aid power calculations for upcoming trials.

### 4.2. Large Dataset Analysis

Various research methods have been presented in this Special Issue, including laboratory research and prospective cohort studies. Research groups are making innovative use of large epidemiological datasets to identify relevant associations, such as a Danish group who identified tens of thousands of patients from national COPD databases.

It is important that therapeutic benefits outweigh risks. Eosinophil-mediated COPD—where corticosteroids are recommended—only make up a fifth of patients. Consequently, corticosteroids are overprescribed with the associated adverse effects of delayed neutrophil clearance and impaired immune function, increasing pneumonia risk by 50% [4]. Corticosteroids also cause systemic adverse effects, including obesity, immunosuppression and psychosis. Eosinopenic patients managed with corticosteroids may suffer adverse effects without experiencing a clinical benefit. Given the emphasis placed on endotype-directed management, appreciating adverse effect burden is key to understanding concordance whilst avoiding iatrogenic injury [18].

Due to the role of endogenous glucocorticoids in stress, mood disorders and psychosis are associated with exogenous corticosteroids, albeit normally with systemic use. Using this large dataset, a group of authors identified that patients requiring high-dose ICS were more frequently prescribed antidepressants and admitted to inpatient psychiatric facilities, despite adjusting for previous antidepressant use [27]. These findings build on established associations between low mood and worsening clinical status in COPD; for instance, a 2014 systematic review identified that mood disorders significantly increased the risks of hospitalisation, mortality and length of stay [28]. In future practice, we should be more vigilant regarding these mental–physical interactions, as a deterioration of one potentially worsens the other. Observational cohort studies carry limitations, including an uncertainty regarding patient compliance or indications for issued prescriptions, as well as the inability to draw causality. For instance, psychiatric symptoms could be the result of troubling COPD symptoms experienced by those with severe disease requiring more corticosteroids.

Using the same methods, the group investigated a rarer consequence of corticosteroids: venous thromboembolism (VTE). COPD patients undergoing exacerbations are already at a high risk of VTE due to underlying inflammation, worsened by corticosteroid-associated hypercoagulability. An analysis revealed that patients requiring longer oral corticosteroid therapy experience a higher risk of VTE hospitalisation, despite accounting for confounders [29]. There is likely an underdiagnosis of VTE in COPD patients presenting with respiratory symptoms, posing the question of whether the findings could be the result of misclassification bias from the small number of VTE events. Our next steps should be to remain mindful of inpatient VTE, particularly in high-risk exacerbating COPD patients. On the other hand, the management of comorbidities also has consequences for COPD exacerbation. Similar to steroids, immunosuppressive agents have been used for their anti-inflammatory and immune-modulatory properties in respiratory conditions such as sarcoidosis but have yet to be studied in COPD. Hence, another study identified that COPD patients who were concurrently on methotrexate for comorbidities had fewer exacerbations of COPD at six months [30]. However, dose dependence could not be established; indeed, even with a large study population of over 50,000 this observational method was inadequately powered.

Finally, this Special Issue includes an integrated epigenomic analysis from Taiwan [31]. Whilst tobacco smoking is strongly implicated in COPD development, sources estimate that 45% of COPD patients have never smoked [32]; instead, air pollutants are implicated in progressive emphysematous development. By matching markers of ambient air pollution with participant residential addresses, researchers determined individual exposure to pollution, which was correlated with pulmonary function and cross-sectional imaging. Markers of air pollution were positively correlated with emphysema severity, and a differential multivariable model was produced. A statistical analysis demonstrated that these demographic and exogenous pollutant data were independent drivers of emphysematous COPD but had compounding effects.

The finding that air pollutants contribute to COPD were established, but this study used artificial neural network modelling to develop a differential pentad model capable of predicting emphysema severity in COPD based on anthropometric, lifestyle and geographical inputs. The researchers set clear inclusion and exclusion criteria to distinguish COPD from similar respiratory conditions. This may, however, have excluded eosinophil-dominant COPD that overlaps with asthma, and for which the proposed questions remain unanswered.

This study is an excellent example of supervised machine learning algorithms analysing large datasets to develop predictive models. Similarly, Wang et al. performed data mining to identify distinct phenotypic clusters in bronchiectasis patients, demonstrating the use of clustering analysis to identify distinct endotypes in complex heterogeneous disease [33]. These methods have public health implications for personalised lifestyle interventions and an enhanced diagnosis. A limitation of these methods is the failure to identify confounders. For instance, whilst Wu et al. present low body mass index (BMI) as being a causative risk factor for the decline in pulmonary function, both air pollution and BMI are confounded by socioeconomic determinants of health. In fact, many types of particulate matter and air pollution markers are confounded by vehicular emissions as the common cause.

## 5. The Future of COPD

Future management considerations in COPD are leaning towards patient-specific endotype-driven therapies. Table 1 outlines various identified subgroups of COPD, and potential therapeutic targets presented in this Special Issue. We are filling in the gaps by understanding emerging subgroups, such as the role of iron in systemic inflammation and microRNA in pre-malignancy. Ultimately, the study of COPD is challenging given its heterogeneous nature. By designing future trials using standardised instruments, making use of next-generation genotyping and prioritising critical ERS outcomes, we can target homogenous groups within the wide spectrum of COPD.

## Figures and Tables

**Table 1:** Subgroups within COPD with potential management considerations prompted by this Special Issue. Table adapted from Turner et al. [5].

Subgroup	Established Treatment	Future Management Considerations
Frequent exacerbator	LABA, LAMA, ICS, roflumilast, macrolides	Optimisation of comorbid physical and mental health conditions [27]
Chronic bronchitis	Roflumilast, mucolytics	Use of CFTR modulators [13]
Emphysema	Lung volume reduction surgery	Correction of miR overexpression [8]
Type 1 respiratory failure	Long-term oxygen therapy	Increased vigilance for VTE in acute illness [29]
Type 2 respiratory failure	Domiciliary NIV	Consideration of comorbidities such as OSA/ORRF [21]
Eosinophilic COPD	Steroids	Identification of distinct microbiome in eosinophil-predominant COPD [15] Investigation of immunomodulatory alternatives to steroids [30]
Bronchiectasis	Targeted antibiotics, chest physiotherapy	Identify severity clusters using biomarkers, to stratify follow-up and hospitalisation [33]
α-1 antitrypsin deficiency	LABA, LAMA, ICS	α-1 antitrypsin augmentation therapy [17]
Subgroups requiring further study		
Biomass and pollutant COPD	Removal of pollutant exposure	Use of predictive machine-learning to target individuals at greatest risk of pollutant-induced emphysema [31]
Premalignant COPD	Smoking cessation	Monitoring markers of oxidative stress and miR genotyping for precision-based chemotherapy [10]
Iron-deficient COPD	IV iron replacement	Monitoring hepcidin as a marker for non-anaemic iron deficiency [23]
Antimicrobial-resistant COPD	Targeted antibiotics based on culture sensitivities	Use of colour charts to determine commencement of antibiotics [14]

Key-LABA: long-acting β-agonists; LAMA: long-acting muscarinic antagonists; ICS: inhaled corticosteroids; HRQoL: health-related quality of life; CFTR: cystic fibrosis transmembrane receptor; miR: microRNA; VTE: venous thromboembolism; NIV: noninvasive ventilation; OSA/ORRF: obstructive sleep apnoea/obesity-related respiratory failure; COPD: chronic obstructive pulmonary disease; IV: intravenous.
